# Neural Basis of Depression Related to a Dominant Right Hemisphere: A Resting-State fMRI Study

**DOI:** 10.1155/2018/5024520

**Published:** 2018-06-05

**Authors:** Mi Li, Hongpei Xu, Shengfu Lu

**Affiliations:** ^1^Laboratory of Intelligent Science & Technology, Department of Automation, Faculty of Information Technology, Beijing University of Technology, Beijing 100024, China; ^2^Beijing International Collaboration Base on Brain Informatics and Wisdom Services, Beijing 100024, China; ^3^Beijing Advanced Innovation Centre for Future Internet Technology, Beijing University of Technology, Beijing 100024, China

## Abstract

**Background:**

In the past, studies on the lateralization of the left and right hemispheres of the brain suggested that depression is dominated by the right hemisphere of the brain, but the neural basis of this theory remains unclear.

**Method:**

Functional magnetic resonance imaging of the brain was performed in 22 depressive patients and 15 healthy controls. The differences in the mean values of the regional homogeneity (ReHo) of two groups were compared, and the low-frequency amplitudes of these differential brain regions were compared.

**Results:**

The results show that compared with healthy subjects, depressive patients had increased ReHo values in the right superior temporal gyrus, right middle temporal gyrus, left inferior temporal gyrus, left middle temporal gyrus, right middle frontal gyrus, triangular part of the right inferior frontal gyrus, orbital part of the right inferior frontal gyrus, right superior occipital gyrus, right middle occipital gyrus, bilateral anterior cingulate, and paracingulate gyri; reduced ReHo values were seen in the right fusiform gyrus, left middle occipital gyrus, left lingual gyrus, and left inferior parietal except in the supramarginal and angular gyri.

**Conclusions:**

The results show that regional homogeneity mainly occurs in the right brain, and the overall performance of the brain is such that right hemisphere synchronization is enhanced while left hemisphere synchronization is weakened. ReHo abnormalities in the resting state can predict abnormalities in individual neurological activities that reflect changes in the structure and function of the brain; abnormalities shown with this indicator are the neuronal basis for the phenomenon that the right hemisphere of the brain has a dominant effect on depression.

## 1. Introduction

Previous studies revealed a link between the left and right cerebral hemispheres and depression, providing a better understanding of the neuropathology of depression. Liotti and Tucker's results of a side-by-side simple response task showed that the left visual field response is impaired in healthy people with a depressive mood and in mild unipolar depressive patients, while the right visual field of healthy people with a depressive mood and in mild unipolar depressive patients did not show a slow response time for the viewing material [1]. A related EEG study in emotion recognition tasks also found that higher right frontal activity was associated with negative emotions and higher left frontal activity was associated with positive emotions [2]. When the healthy subjects were induced to experience negative emotions, the alpha power of the right frontal lobe was more inhibited than the left frontal lobe was, whereas in cases of positive emotions, the asymmetry of alpha power in the relevant region showed opposite results. These findings indicate that individuals with higher right frontal lobe activity have stronger negative emotions than individuals with higher left frontal lobe activity have [3–5]. The above findings discuss the relationship between the left and right hemispheres and emotions. The right frontal activity is enhanced in depressive patients or healthy subjects who were induced to experience depressive emotions.

There are also differences in information processing between the left hemisphere and the right hemisphere. The information processing of the left hemisphere is characterized by sequence, analysis, and logic; the information processed by the right hemisphere is parallel, holistic, and intuitive [6]. Based on the differences between left and right brain information processing, many experiments were designed to determine the characteristics of different hemispheric cognitive functions. The tachistoscopic visual half-field technique [7] and the dichotic listening technique [8] have been used to examine the effects of each hemisphere in visual and auditory processing. Bruder et al. used dichotic listening and visual half-field tasks to detect the perceptual asymmetry of depressive patients. The results showed that the perceived asymmetry of depressive patients was consistent with the hypothesis of right hemisphere dysfunction [9]. Similarly, in a study by Kaprinis et al., 26 patients with bipolar disorder were subjected to a dichotic test during a manic phase and after recovery; the manic phase showed a significant left ear (right hemisphere) advantage, and after recovery, patients shifted to a right ear advantage. These results show that a characteristic of manic disorder is the overactivation of right hemisphere function [10]. EEG and neuroimaging studies have also been extensively used in the study of hemispheric lateralization in depression. Flor-Henry et al. used EEG to analyze the differences between the left and right hemispheres of subjects in a resting state and during language and spatial cognitive tasks, and the results showed that negative emotions and depression were associated with relatively high activity in the frontal cortex of the right hemisphere [11]. Relevant neuroimaging studies reported that patients with unipolar depression are characterized by metabolic decline in the left brain and metabolic enhancement in the right brain [12]. The severity of depression is positively correlated with the metabolic enhancement of the right hemisphere [13]. The high activity in the right frontal lobe and the relatively low activity in the left frontal lobe in depressed patients have been confirmed in most surveys, with only a few exceptions [14]. A functional right hemisphere can make individuals overcome negative emotions and prevent the development of depression [15].

Bruder et al. systematically reviewed evidence of left and right brain asymmetry in electrophysiological (EEG and event-related potential), behavioral (dichotic and visual perceptual asymmetry), and neuroimaging (PET, MRI, and NIRS) and discussed that individual differences in left and right brain function measurements in patients with depression are related to the clinical diagnosis of subtypes of depression, anxiety disorders, and clinical response to antidepressant drugs or cognitive behavioral therapy [16]. Bruder et al. found that depressed patients with an anxiety disorder exhibited a frontal alpha asymmetry, whereas nonanxious depressed patients did not show this asymmetry [17]. Gold et al. found that frontal asymmetry was significantly associated with the degree of anxiety rather than the severity of depression [18]. Some studies have also found that the parietal region of depressed or previously depressed individuals has an opposite asymmetry compared to the frontal lobe (i.e., the right hemisphere has less activity than the left hemisphere) relative to the subjects of no depression [19, 20]. However, right parietal lobe activity of depressed patients with an anxiety disorder increases or offsets relatively less right parietal lobe activity in adolescents and adults [21]. Alpha asymmetry in patients with major depression and comorbid anxiety indicates that the right side has more activity than the left parietal lobe has, whereas patients with “pure” depression show less activity on the right parietal lobe [16]. It shows that the activity of the parietal lobe is related to depression and whether there is comorbid anxiety.

Previous studies have found abnormal lateralization of right and left hemisphere activity or function in depressive disorders, but the literature does not provide a detailed description of the neurological basis of depression associated with the high activity in the right hemisphere. Zang et al. proposed a regional homogeneity method to understand brain function in the resting state. Abnormalities are associated with temporal changes in neural activity of local areas and can be used to detect abnormal activity throughout the brain [22]. High ReHo values indicate that high synchronous oscillations of neurons in ALFF are directly related to the intensity of spontaneous neural activity in energy metabolism at resting state [23]. Kong et al. compared the differences in ReHo and low-frequency amplitude (ALFF) in brain regions before and after treatment with electroconvulsive therapy (ECT) in elderly patients with major depressive disorder (MDD); results show that ECT may affect brain function in elderly patients with MDD at resting state [24]. Yao et al. compared the ReHo of bipolar depression (BD), unipolar depression (UD), and healthy controls (HC) in the whole brain. The results show that the left frontal cluster (LFC) had significant differences among the three groups of subjects and the left temporal cluster (LTC) has a significant difference between BD and UD; the cuneus may provide neurological signs of depression in patients with BD and UD [25]. Peng et al. studied the ReHo of whole brain fMRI in patients with major depression and healthy controls at rest, and the results show that the ReHo values of the left thalamus, left temporal lobe, left cerebellar posterior lobe, and bilateral occipital lobe in patients with depression were significantly lower than in healthy subjects, suggesting abnormal activity in these brain regions [26]. Liu et al. found that the ReHo values of the right insula and left cerebellum of patients with MDD and high-risk MDD were significantly lower than those of healthy controls [27]. Guo et al. studied ReHo values in the brain regions of the first-time, initial, short-term, and treatment-responsive depression patients at resting state to detect abnormal marginal cortical networks in the MDD; the results show that the marginal cortical networks in first-time, initial, short-term, and treatment-responsive MDD patients are abnormal [28]. Wu et al. compared ReHo values of resting-state fMRI in treatment-refractory depression (TRD), depressed but not treatment-refractory (NDD), and healthy controls; the results show that compared with the healthy control group, the ReHo values within temporolimbic structures in the two depression groups were large and the ReHo values in the frontal, parietal, and posterior fusiform cortices and caudate were small; patients with TRD have more ReHo-modified brain regions than those with NDD have, demonstrating the feasibility of using ReHo as a research and clinical tool to monitor persistent cerebral dysfunction in depression [29]. Yao et al. used the ReHo method to explore the brain activity characteristics of MDD patients in resting state; the results showed that the severity of anxiety was positively related to ReHo in the right insula; the degree of cognitive impairment is positively correlated with ReHo in the right frontal cortex and left anterior cingulate gyrus; late severity was positively associated with ReHo in the right posterior cingulate gyrus and the right insula; the severity of sleep disorders was positively correlated with ReHo in the dorsomedial anterior cingulate gyrus; and desperate severity is positively correlated with right ventral anterior cingulate gyrus and right inguinal reanimation [30]. Liang et al. studied changes in brain activation patterns in bipolar disorder (BD) and unipolar depression (UD) patients. The results show some overlap in ReHo between UD and BD groups; however, significant differences in the thalamus of BD can assist in detecting functional defects and distinguish clinical and pathophysiological signs of BD and UD [31].

To explore the highly activated neural basis of the right hemisphere of depression, this paper analyzed and compared ReHo values across the entire brain area between patients with depression and healthy controls and extracted the amplitude of low-frequency fluctuation (ALFF) of the brain areas that showed significant differences in ReHo measures between depressed patients and the control group to verify our guess.

## 2. Materials and Methods

### 2.1. Subjects

A total of 22 depressed patients and 15 healthy controls were recruited from the Anding Hospital in Beijing, China. All participants' diagnostic evaluations were conducted using DSM-IV-based Mini International Neuropsychiatric Interview 6.0 (MINI 6.0) by clinically trained and experienced evaluators [32]. Physiological reports were performed on MDD and HC groups using a nine-item patient health questionnaire (PHQ-9). The demographic and clinical characteristics of the subjects are given in Table 1. Depressive patients suffer from severe anxiety (generalized anxiety disorder > 10) in this paper. Exclusion criteria were the following: (1) depressive patients with a history of any manic episode or any major mental illness on axis I or axis II, (2) simultaneously suffering from a severe disease or primary nervous system disorder, (3) a history of loss of consciousness due to head injury, (4) abuse or dependence on alcohol or other substances, and (5) MRI contraindications.

### 2.2. MRI Data Acquisition

The fMRI data were collected by a Siemens Trio Tim 3T scanner. Functional data were scanned by a gradient plane echo pulse sequence. The head of the subject was fixed with a sponge pad to reduce the effects of head movement on the experimental results. During the resting-state scan, subjects kept their eyes closed, were sober, and relaxed their bodies. The scanning parameters were as follows: TR (scanning repetition time) = 2000 ms, TE (echo time) = 30 ms, FOV (field of view) = 224 mm × 224 mm, FA (rotation angle) = 90°, matrix = 64 × 64, gap (spacing of layers) = 0 mm, a total of 33 layers of axial images covering the whole brain, and a voxel size = 3.5 mm × 3.5 mm × 3.5 mm. The scan time of each subject was 860 seconds, 860 functional images frames were collected, and the corresponding BOLD signal contained 430 time points.

### 2.3. Image Preprocessing

Image preprocessing was completed on a MATLAB platform using SPM12 Software (Wellcome Trust Center for Neuroimaging, London, UK; http://www.fil.ion.ucl.ac.uk/spm/ext/) and REST toolkit. To eliminate the instability of the magnetic field at the beginning of the scan, the data from the first ten time points were removed, and only the remaining 420 time points were processed and analyzed. The main steps of image preprocessing are described below. (1) Slice-timing correction was performed to correct the layer time to eliminate the error caused by the removal of sequences at the beginning of the scan. (2) Realignment was performed to correct the differences in head translation or rotation during data collection. If the degree of translation or rotation of the subjects was within a certain range, the artifacts and errors produced by the head movement could be eliminated by realignment. If the degree of translation or rotation was too large and could not be eliminated by software corrections, the subjects were removed. In this study, the standard translation was less than 2.0 mm and the rotation angle was less than 2.0°; ultimately, four subjects were excluded. (3) Coregistration was performed, where the images obtained at different times were aligned in space and time. (4) Data were normalized, where different subjects' fMRI images were registered to the MNI standard brain anatomical template to correct for individual differences in brain structure. The specific steps were as follows: first, the 3D structural image of each subject was matched with the functional image of the resting state, and then the 3D structural image was divided into gray matter, white matter, and cerebrospinal fluid. The parameters of the structural image for the standard template were written into the aligned functional image; at the same time, if the functional image needed to be resliced, the voxel size of the reslice was 3 × 3 × 3 mm^3^. (5) Detrending was performed to remove the linear trend produced by a rise in temperature due to the machine working or adaptation of the subject over time. (6) The resting-state BOLD signal was filtered using a filter with a frequency band of 0.01 to 0.1. This frequency range mainly reflects the spontaneous activity of nerve cells. (7) Smoothing was performed to reduce the spatial structural differences between subjects, improving the signal-to-noise ratio. The spatial normalization of the image was carried out with 4 mm full width at half-maximum Gaussian space smoothing, which improves the validity of the statistical tests. (8) ReHo and ALFF were calculated, where the brain data of the subjects were calculated after the abovementioned processing steps. It should be noted that the smoothing was done after the calculation of ReHo. Because the consistency of a voxel and its surrounding voxels will increase after smoothing, the ReHo analysis must first calculate the Kendall harmony coefficient of each voxel and then the ReHo diagram should be smoothed to eliminate the standardization of small errors and improve the signal-to-noise ratio (using 4 × 4 × 4 mm Gaussian kernel). The calculation of ALFF needs to follow the smoothing step, and then the time series of each voxel in the image should be converted into a frequency range using a Fourier transform to obtain a frequency power spectrum, where the peak area can be regarded as the amount of energy. The power spectrum of the signal with a frequency range of 0.01–0.1 Hz was squared to obtain ALFF.

### 2.4. Regional Homogeneity (ReHo) Analysis

Regional homogeneity is a brain imaging method based on functional magnetic resonance imaging data. Assuming that the temporal sequence of the selected voxel is similar in time to the adjacent 26 voxels, an increase in the indicator implies that the local neuronal activity tends to be synchronized. Conversely, this metric can show disorders over time. ReHo is calculated by Kendall's concordance coefficient (KCC). The formula for calculation is as follows:

(1) $$\begin{array}{c} W=\frac{\sum_{i=1}^{n}{\left(R_{i}\right)}^{2}-n{\left(\bar{R}\right)}^{2}}{\left(1/2\right)K^{2}\left(n^{3}-n\right)}, \end{array}$$

where *W* represents Kendall's concordance coefficient of a specific point, which ranges from 0 to 1, indicating the regional homogeneity of the BOLD signal from weak to strong, respectively; *n* represents the number of time points, which in this paper was 420; *K* represents the total number of specific voxels and the surrounding voxels (which is 7,19,27), and in this paper *K* was 27; *R*_*i*_ is the total number of levels of voxels of each voxel at the *i*th time point; and $\bar{R}$ is the average of *R*. The closer the KCC value is to 1, the higher the synchronization of local neural activity in the brain and the more orderly the neural activity is.

### 2.5. Amplitude of Low-Frequency Fluctuation (ALFF)

Electrophysiological studies show that the generation of low-frequency oscillations may be due to spontaneous neuronal activity. This spontaneous neuronal activity is physiologically significant, reflecting the rhythmic activity patterns a brain region produces by interacting with its connected brain regions. Thus, there is reason to believe that ALFF can serve as an indicator of brain characteristics. Low-frequency oscillations (LFO) are mainly observed with low-frequency amplitudes, and the low-frequency amplitude range selected for this study was between 0.01 and 0.1 Hz. The low-frequency amplitude was calculated as follows:

(2) $$\begin{array}{c} ALFF=\underset{K:f_{k}\in \left[0.01,\;0.1\right]}{\sum }\sqrt{\frac{a_{k}{\left(f\right)}^{2}+b_{k}{\left(f\right)}^{2}}{N}}. \end{array}$$

This formula calculation is the sum of the amplitude at a given frequency, where *a*_*k*_ and *b*_*k*_ are coefficients corresponding to different frequencies and *N* is the total number of voxels. In this paper, we did not directly analyze the whole brain ALFF but extracted the low-frequency amplitude (ALFF) of the brain areas that showed significant differences in ReHo measures between depressed patients and the control group. Differences between groups were tested using two-sample *t*-tests.

## 3. Results

### 3.1. Subjects

Twenty-two depressive patients and 15 healthy subjects completed the fMRI scan. One patient and 1 healthy subject were excluded due to incomplete data at the functional imaging time point. Four patients were excluded due to excessive head movements, and 1 patient was excluded due to a lack of spatially standardized results, leaving 16 patients and 14 healthy subjects.

### 3.2. ReHo: Depression versus Healthy Subjects

**Figure 1:**
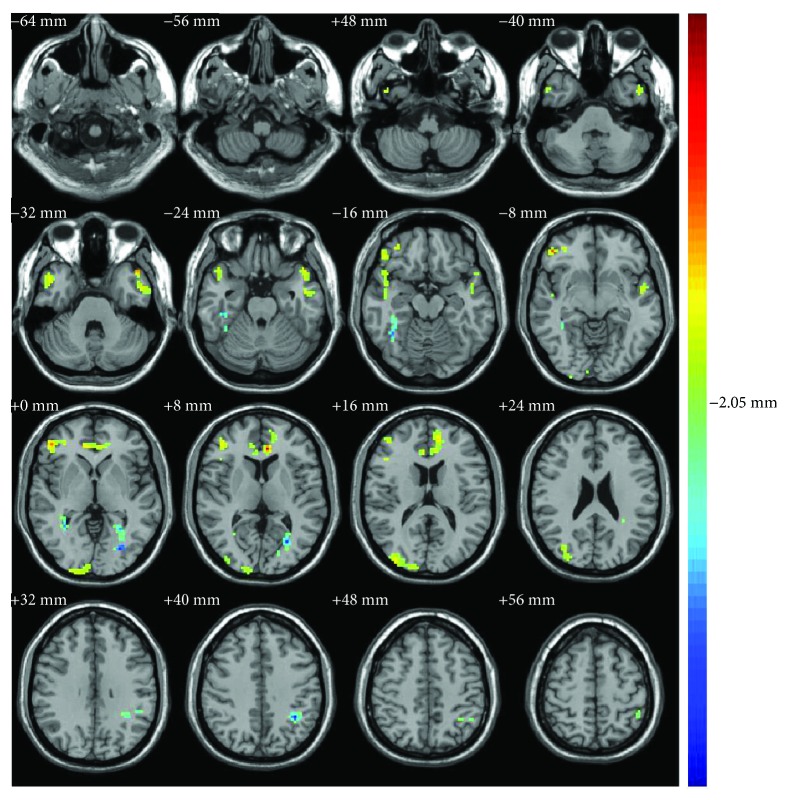
Brain regions with increased/decreased regional homogeneity (ReHo) (patients versus controls, independent *t*-test, *P* < 0.05, cluster size > 8 voxels; the left side of each brain image in the figure shows the right brain and the right side shows the left brain, and the color from blue to red indicates that the significant difference is from small to large).

The ReHo brain data of the depression group and the healthy control group were analyzed by two-sample *t*-tests [26, 29]. The results showed that compared with the healthy control group, the brain BOLD signal of the depression group in the resting state was significantly different. As shown in Figure 1 and Table 2, the ReHo values were higher in depressed patients in the right superior temporal gyrus, right middle temporal gyrus, left inferior temporal gyrus, left middle temporal gyrus, right middle frontal gyrus, triangular part of the right inferior frontal gyrus, orbital part of the right inferior frontal gyrus, right superior occipital gyrus, right middle occipital gyrus, bilateral anterior cingulate, and paracingulate gyri, and values were reduced in the right fusiform gyrus, left middle occipital gyrus, left lingual gyrus, and left inferior parietal except for the supramarginal and angular gyri. In Figure 1, the colored area represents the brain regions with differences in ReHo between patients with depression and healthy subjects; the differences include both the increased ReHo brain area and the reduced ReHo brain area of depression.

**Figure 2:**
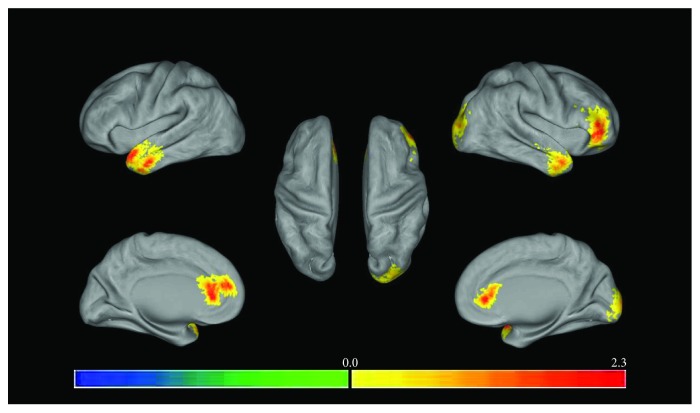
Regional homogeneity (ReHo) where depression group > control group (independent *t*-test, *P* < 0.05, cluster size > 8 voxels; the left half of the figure represents the left brain and the right half of the figure represents the right brain, and the color from blue to red represents that the significant difference is from small to large).

**Figure 3:**
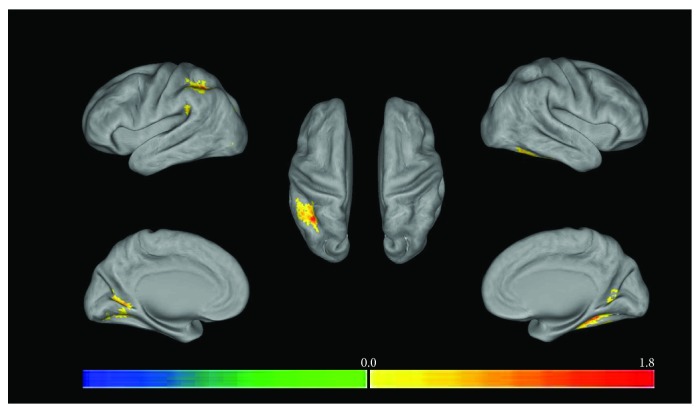
Regional homogeneity (ReHo) where depression group < control group (independent *t*-test, *P* < 0.05, cluster size > 8 voxels; the left half of the figure represents the left brain and the right half of the figure represents the right brain, and the color from blue to red represents that the significant difference is from small to large).

In the multibaseline 3D graph (the multifiducial map gives a smoother map and the best estimate of spatial localization), Figure 2 shows regions where the ReHo value in the depressed patients was greater than in the healthy control group, and Figure 3 shows regions where ReHo values were greater in healthy controls than in the depressed patients. Finally, the low-frequency amplitude (ALFF) of the brain regions with significant differences in ReHo measures between the depression group and the healthy control group was extracted for two-sample *t*-tests. The results are shown in Table 3. The ALFF for the depression group was higher in the left middle temporal gyrus, while the other brain regions were not significantly different from the controls.

## 4. Discussion

In this study of resting-state fMRI, we used the regional homogeneity (ReHo) method to analyze fMRI data. This indicator can measure the time consistency of local blood oxygen level-dependent signals and reflect the time consistency of neural activity. The results show that there are synchronal abnormalities in some brain areas of depressive patients in the resting state.

Compared with the healthy subjects, we found that the ReHo value in the depression group was higher in the right middle frontal gyrus, triangular part of the right inferior frontal gyrus, and orbital part of the right inferior frontal gyrus; the frontal lobe region showed the most intense activation (the highest peak area was located in the orbital part of the inferior frontal gyrus, *t* = 6.45). Frontal lobe activity is related to the characteristics of depressive patients, including functional abnormalities, emotional adjustments, and cognitive controls [19]. To date, frontal lobes have been identified as one of the most consistent areas associated with MDD [33]. Yao et al. found that the ReHo value of major depressive patients with anxiety disorder was reduced in the left middle frontal gyrus, but no abnormalities were found in the right middle frontal gyrus [30, 34]; however, this paper has not completely ruled out the effect of antidepressants on brain activity in depressed patients which is likely to cause different findings with our paper.

The ReHo values in the depression group of the current study were increased in the right superior temporal gyrus, right middle temporal gyrus, left inferior temporal gyrus, and left middle temporal gyrus, as well as in the superior temporal gyrus. The right superior temporal gyrus is involved in the perception of facial expressions of emotion [35, 36] and the left middle temporal gyrus, which is associated with text and language processing. Wu et al. have found results consistent with this study. Additional correlation analysis showed that anxiety symptoms were associated with dysfunction of the temporal lobe brain area [30, 37]. For example, other studies have reported that the ReHo value in depressive patients is reduced in the left superior temporal gyrus and the left temporal lobe [26], which is inconsistent with the results of this study. The paper's Hamilton Anxiety Scale (HAMA) < 14 indicates that patients with major depression may have anxiety but no obvious or no symptoms of anxiety; the statistical test results show that there is no significant difference in anxiety symptoms between patients with depression and healthy subjects, and our depressive patients have severe anxiety, indicating that differences of results may be related to anxiety.

The ReHo value of the depression group was higher in the right superior occipital gyrus and right middle occipital gyrus and lower in the left lingual gyrus, bilateral anterior cingulate gyrus, and paracingulate gyri. These areas belong to the occipital lobe, and most of the functional areas of the occipital lobe are related to visual signal coding. The middle occipital gyrus participates in visual processing; the right anterior cingulate and paracingulate gyri, as well as the fusiform gyrus, as part of the marginal system are connected to the amygdala, orbital frontal cortex, and hippocampus and participate in the emotional system, and the left anterior cingulate and paracingulate gyri are responsible for the decision-making process. In a study by Peng et al. [26], the ReHo values of major depressive patients without anxiety were reduced in bilateral occipital lobes, and there were both increased and reduced areas in the current study; the reason for this difference, as described above, may be related to whether or not the depression suffers from anxiety.

The ReHo value of the depression group was reduced in the left inferior parietal, except the supramarginal and angular gyri; this area belongs to the parietal lobe. An EEG study found that patients with major depression and comorbid anxiety showed alpha asymmetry which indicates that the right side has more activity than the left parietal lobe has, whereas patients with “pure” depression show less activity on the right parietal lobe [16]. Bruder et al. reviewed the use of behavioral, EEG, and ERP measurement methods to indicate that there may be defects in right parietal lobe function in depressed patients, especially when dealing with emotional information [16].

The amplitude of low-frequency fluctuation (ALFF) is also commonly used as an indicator of abnormal brain activity. In this study, the amplitude of low-frequency fluctuation of the brain regions with significant differences in ReHo values between the depression group and the healthy subject group was extracted for two-sample *t*-tests. The results showed that the depression group ALFF was higher in the left middle temporal lobe, while the other brain regions were not significantly different between groups. The value of ALFF can directly reflect the strength of the brain's neural activity, and ReHo is an indirect measurement. Whether ReHo is enhanced or weakened indicates that the neural activity in a local area is synchronization abnormalities and the region's collaboration capacity is abnormal. Although the efficiency of the brain can decline, it is still able to complete its corresponding physiological functions. The synchronization abnormalities of local nerve activity above a certain limit will be expressed as neural activity abnormalities, and, at this point, the brain cannot complete its corresponding physiological functions. Although abnormalities in regional homogeneity cannot directly represent neural activity abnormalities, the regional homogeneity of a region with abnormal neural activity must be abnormal, which means that regional homogeneity can predict individual neural activity. From the regional homogeneity results, we found that abnormal areas occurred mainly in the right brain in depressed patients, and the overall brain synchronization performance was enhanced in the right hemisphere and attenuated in the left hemisphere. Based on these results, we can predict neurological activity trends in these patients' brains; that is, the spontaneous nerve activity in ReHo-enhanced brain regions in resting states may be enhanced. In contrast, spontaneous nerve activity in the brain area with ReHo attenuation may be diminished. The amplitude of low-frequency fluctuation (ALFF) of the brain regions with significant differences in ReHo values between the depression group and the healthy control group increased in the left temporal gyrus, indicating that the spontaneous nerve activity in this region was enhanced, thus validating our hypothesis. Furthermore, according to this trend of right hemisphere enhancement and left hemisphere attenuation of ReHo values, we can predict that the spontaneous nerve activity in the resting state of the brain as a whole shows right hemisphere enhancement and left hemisphere attenuation.

Related studies suggest that the physiological activation of the right hemisphere of the brain in depressive patients is accompanied by functional decline and deficiencies. The additional physiological activity in the right hemisphere is to overcome functional barriers. To be able to properly complete corresponding functions, the right brain of depressive patients must increase its activity level (but the result is futile). The increase and decrease of ReHo suggest that neuronal activity is disturbed, indicating that the structure and function of the corresponding brain area are changed. Abnormalities mainly occur in the right hemisphere, which is consistent with the previous studies showing the dominant role of the right hemisphere in depression. Regional homogeneity can predict abnormalities in neurological activity and reflect changes in the structure and function of the brain in the resting state. Abnormalities in this indicator are the neural basis of the phenomenon that the right hemisphere of the brain has a dominant effect on depression.

## Figures and Tables

**Table 1:** Demographic and clinical characteristics of depression patients and healthy controls.

Variables (mean ± SD)	Depression	Healthy controls	*t*/*χ*^2^	*P* value	Cohen's *d*
	(*n* = 16)	(*n* = 14)			
Gender (M : F)	9 : 7	7 : 7	0.12	0.73	
Age (years)	27.38 ± 7.62	30.43 ± 8.50	1.04	0.31	−0.38
Age range	16–43	23–47			
Education level (years)	13.06 ± 3.62	15.00 ± 4.30	1.34	0.19	−0.49
PHQ-9	16.63 ± 5.33	3.29 ± 2.79	8.41	0.00	3.14

PHQ-9: 9-item patient health questionnaire.

**Table 2:** Brain areas with significant ReHo differences between depression patients and controls.

Brain regions	Side	Brodmann areas	Coordinates (MNI)			*k*	*t* statistic
			*x*	*y*	*z*		
Patients > control							
Middle frontal gyrus	Right	45/46	45	45	12	46	3.51
Inferior frontal gyrus, triangular part	Right	10/45	45	42	3	40	4.24
Middle frontal gyrus, orbital part	Right	47	48	42	−6	52	6.45
Temporal pole: superior temporal gyrus	Right	38	48	18	−18	45	3.44
Temporal pole: middle temporal gyrus	Right	20	45	12	−27	17	3.37
Temporal pole: middle temporal gyrus	Left	38	−48	18	−33	23	3.96
Inferior temporal gyrus	Left	20	−51	−6	−27	44	3.66
Superior occipital gyrus	Right	17	15	−102	6	33	2.84
Middle occipital gyrus	Right	19	36	−90	12	56	3.31
Anterior cingulate and paracingulate gyri	Left	24	−6	36	9	61	5.36
Anterior cingulate and paracingulate gyri	Right	32	12	39	3	44	3.52
Patients < control							
Fusiform gyrus	Right	37	33	−54	3	48	−3.67
Middle occipital gyrus	Left	19	−27	−78	0	8	−3.81
Lingual gyrus	Left	18	−18	−78	3	8	−2.05
Inferior parietal, except supramarginal and angular gyri	Left	40	−36	−52	42	57	−4.43

**Table 3:** ALFF differences in brain areas with significant ReHo differences between depression patients and controls.

Brain regions	Side	ALFF			
		Depression	Control	*t*	*P*
Patients > control					
Middle frontal gyrus	Right	0.83 ± 0.08	0.85 ± 0.07	−0.64	0.53
Inferior frontal gyrus, triangular part	Right	0.72 ± 0.05	0.75 ± 0.09	−1.11	0.28
Middle frontal gyrus, orbital part	Right	0.82 ± 0.09	0.88 ± 0.12	−1.42	0.17
Temporal pole: superior temporal gyrus	Right	0.95 ± 0.14	0.99 ± 0.13	−0.88	0.38
Temporal pole: middle temporal gyrus	Right	0.57 ± 0.09	0.55 ± 0.07	0.45	0.66
Temporal pole: middle temporal gyrus	Left	0.69 ± 0.11	0.62 ± 0.08	2.19	0.04
Inferior temporal gyrus	Left	0.79 ± 0.27	0.77 ± 0.05	0.83	0.42
Superior occipital gyrus	Right	1.04 ± 0.12	1.05 ± 0.13	−0.24	0.81
Middle occipital gyrus	Right	1.04 ± 0.13	1.07 ± 0.15	−0.49	0.63
Anterior cingulate and paracingulate gyri	Left	0.78 ± 0.06	0.82 ± 0.05	−2.02	0.54
Anterior cingulate and paracingulate gyri	Right	0.92 ± 0.11	0.94 ± 0.07	−0.61	0.55
Patients < control					
Fusiform gyrus	Right	1.10 ± 0.08	1.09 ± 0.08	0.54	0.60
Middle occipital gyrus	Left	1.09 ± 0.12	1.09 ± 0.13	−0.13	0.90
Lingual gyrus	Left	1.15 ± 0.17	1.10 ± 012	0.86	0.40
Inferior parietal, except supramarginal and angular gyri	Left	0.98 ± 0.13	1.05 ± 0.09	−1.73	0.10

## Data Availability

Since these data relate to personal, private information of patients and health subjects, there are ethical restrictions. All original data referred to in the paper will be made available upon request to the corresponding author.
